# Analytical and economical optimization of a glucose method with immobilized enzymes

**DOI:** 10.1155/S1463924683000462

**Published:** 1983

**Authors:** I. Andersen, S. Hannibal

**Affiliations:** 1 Department of Clinical Chemistry Copenhagen County Hospital in Herlev University of Copenhagen Herlev DK-2730 Denmark; 2 Biomedical Laboratory Copenhagen County Hospital in Herlev Herlev DK-2730 Denmark


Journal of Automatic Chemistry, Volume 5, Number 4 (October–December 1983), pages 188–192

### Analytical and economical optimization of a glucose method with immobilized enzymes

**I. Andersen**
*Department of Clinical Chemistry, Copenhagen County Hospital in Herlev, University of Copenhagen, DK-2730 Herlev, Denmark*

**and S. Hannibal**
*Biomedical Laboratory, Copenhagen County Hospital in Herlev, DK-2730 Herlev, Denmark*

### Introduction

The use of immobilized enzymes for substrate determinations has a number of unique advantages over the use of enzymes in free solutions:

(1) Immobilized enzymes are water insoluble and can therefore be recovered at the end of the reaction and be reused many times.
(2) Enzymes immobilized to insoluble matrices bear a closer resemblance to enzymes *in vivo* than do free enzymes in dilute aqueous solutions. The stability is therefore considerably enhanced.

So, in principle, the use of immobilized enzymes for analytical purposes is economically attractive; however, until now they have not been employed in clinical chemistry as often as might be expected. Perhaps the reason is that the advantages can very easily disappear if the analytical system is misused, for example by cleaning it with a solution with an extreme pH, with an organic solvent or a detergent. The use of immobilized enzymes has been limited to integrated continuous-flow systems, where control of valves and cleaning is conducted with a computer.

Because glucose is one of the most frequently requested quantities in clinical chemistry laboratories, it is important that the method is optimized both analytically and economically. This paper discusses several modifications to the method.

The original method, 'Glucose (Hexokinase) Bound Enzyme Method for AA II systems, SE 40046FD9' (Technicon Instruments Corporation, Tarrytown, New York 10591, USA), is modified with respect to pre-treatment and stability of specimen, type of sample material, durability of the enzyme reactor and reduction of the reagent consumption. If the glucose channel is a part of a multichannel analyser (for example an SMA), an electronic controller is constructed, which by means of solenoid valves (PSV-3, Pharmacia Fine Chemicals, Uppsala, Sweden) both protects the enzyme reactor and reduces the reagent consumption by recirculation.

### Experimental

### The analytical system

The system is illustrated in figure 1; details concerning the manifold are given, together with Technicon accessory numbers, in figure 2.

With a production rate of 100 determinations/h and a sample-to-wash ratio of 2 : 1, the carry-over, *c*, is estimated to $\leqslant 3{\cdot}0\%$:

$$c=\left[\frac{L_1-L_3}{H_3-L_3}\right]\times 100,$$

where $H_1$, $H_3$, $L_1$ and $L_3$ are measured concentrations of high and low level analytes.

### Reagents

*Diluent*
All samples are automatically diluted six times before dialysing with 0·154 mol/l sodium chloride with 0·1 ml 30% Brij-35 per 100 ml.

*Recipient*
On the recipient side of the C membrane the glucose from the sample is mixed with the working reagent: ATP, NAD, $MgCl_2$ and $Na_2Mg$ EDTA in a Pipes buffer. The reagent is in accordance with the one used in the Technicon method SE4-0046FD9, described by Leon *et al.* [1].

The stability of the reagent is guaranteed to be one week at room temperature. Unchanged results have been found on a control (10 mmol/l) using reagents stored up to one month at room temperature.

### Preparation of samples

Whole blood is taken from the patient's ear lobe into a 20 μl heparinized glass capillary and collected directly into a 400 μl polyethylene tube with 300 μl of a solution containing heparin (100 IU/ml), NaF (0·12 mol/l) and benzoic acid (0·02 mol/l). EDTA plasma and urine can also be used. After closure with the attached airtight cap, the capillary inside is broken by bending the plastic tube once. The tube is then turned upside down and the contents mixed—the material is now ready for analysis. The stability of glucose in this diluent is more than one month at room temperature.

### Modifications

This method differs from the Technicon method with respect to the following:

(1) The specimen used—whole blood/EDTA plasma/urine diluted with heparin, NaF, benzoic acid instead of serum.
(2) The volume rate of sample aspiration into the flow system—0·16 rather than 0·10 μl/min.
(3) The length of the dialyser—24 instead of 6 in.
(4) The production rate—100 rather than 60 determinations/h.

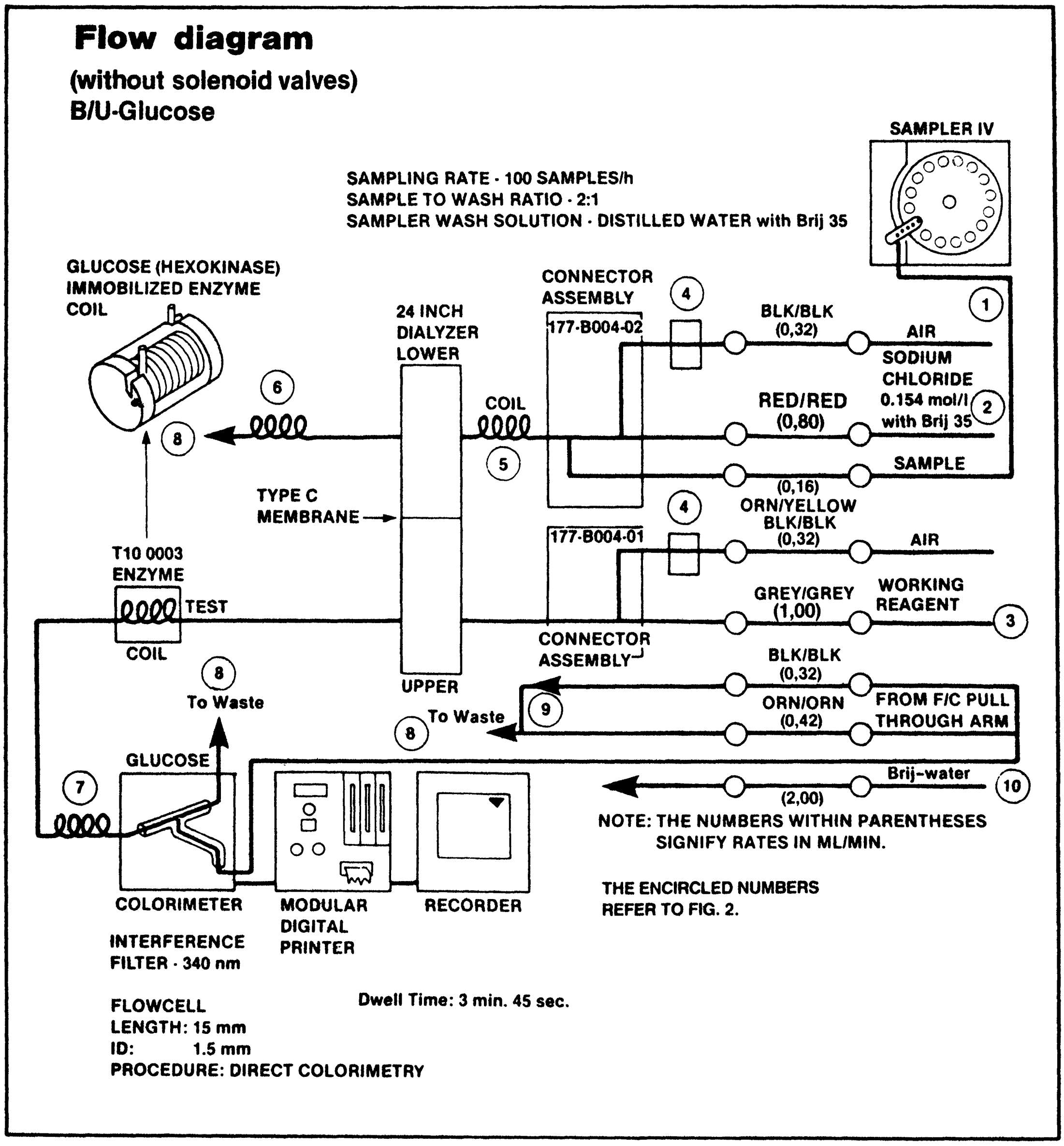

*Figure 1. Flow diagram. The Technicon spare parts numbers are explained in figure 2.*

### *Valves*

When the analyser is of the single-channel type, the three reagent lines are equipped with one-way valves so that the flow can be stopped immediately by unlatching the cover plate and platen of the proportioning pump without back-flush of reagents. So the bubble pattern is undisturbed and it is possible to stop and start analysing without breaking the pattern; this results in a reduction in reagent consumption.

If the analyser is of a multichannel type, another type of valve (solenoid valve, PSV-3, Pharmacia Fine Chemicals, Sweden) is used because it now has several functions. The four valves are connected to a controller.

The solenoid valves secure protection of the enzyme coil when the manifold is cleaned by means of sodium hydroxide (0·2 mol/l); reduction of the reagent consumption by recirculation of the working reagent when the system is in stand-by mode; bypassing the recipient side of the dialyser when the system is in stand-by mode, so that the working reagent remains undiluted when recirculated.

Figure 3 shows the reagent flow when the valves are in the analysing mode, in the stand-by mode and in the wash mode. The valves are connected to a controller (diagrams are available on request from S. Hannibal), which is attached to the sampler on/off button, so that the routine-operation of the analyser is unchanged.

*Figure 2. Explanation of the Technicon spare parts numbers in figure 1.*

| Figure 1 No. | Technicon spare part No. |
|---|---|
| 1 Sample tube | 0·10-116-536-03 |
| 2 Tube for 0·154 mol/l NaCl with Brij-35 1% v/v | 0·35-116-536-08 |
| 3 Tube for working reagent | 0·40-116-536-09 |
| 4 Airbar tube (silicone) | T-116-054301 |
| 5 Coil | 178-G 196-02 10 turns |
| 6 Coil | 178-G 196-03 20 turns |
| 7 Coil | 178-G 196-02H 10 turns |
| 8 Waste | 116-528-01 |
| 9 Three-way nipple | 518-0274-01 |
| 10 Tube for Brij-water (Brij-35 : 1% v/v) | 0·40-116-536-09 |
| All transmission tubes | 116-528-01 |

Nos. 2, 3 and 10 are equipped with one-way valves (556-1045), see text for description.

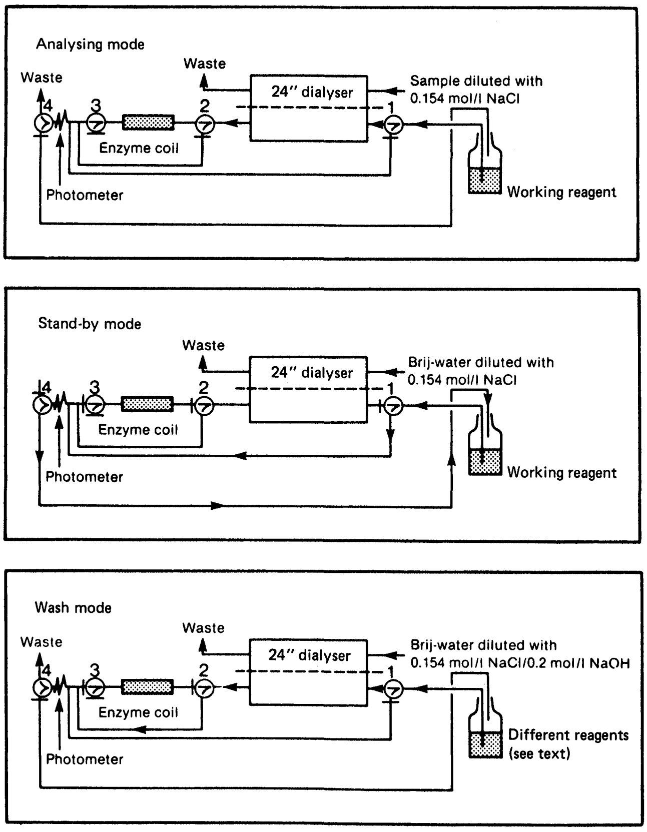

*Figure 3. Reagent flow in the different modes. The reagent flow depends on the position of the solenoid valves which are connected to the controller; the controller is attached to the sampler on/off.*

*Analysing mode (sampler on): circulation of working reagent through valve 1, dialyser; valve 2, enzyme coil; valve 3, photometer; valve 4 and to waste. When the sampler is off the flow continues for X min (the maximum is 9 min), before the valves change to stand-by. (X is the setting of the timer, 'Number of minutes before recirculation', and represents the dwell time from sampling to valve 4.)*

*Stand-by mode (sampler off in X min): recirculation of working reagent through valve 1 (bypassing the dialyser and enzyme coil), photometer, valve 4 and to the origin.*

*Wash mode (stand-by mode with wash button activated on the controller): circulation of working reagent/0·2 mol/l NaOH/working reagent through valve 1, dialyser, valve 2, (bypassing the enzyme coil), photometer, valve 4 and to waste. When the wash button is deactivated, the stand-by mode is established.*

### Linearity

During a three-month period the linearity of the calibration function was tested daily using the same enzyme coil, which was stored at room temperature (see figure 4). The upper limit decreased from 40 mmol/l to 20 mmol/l in that period;

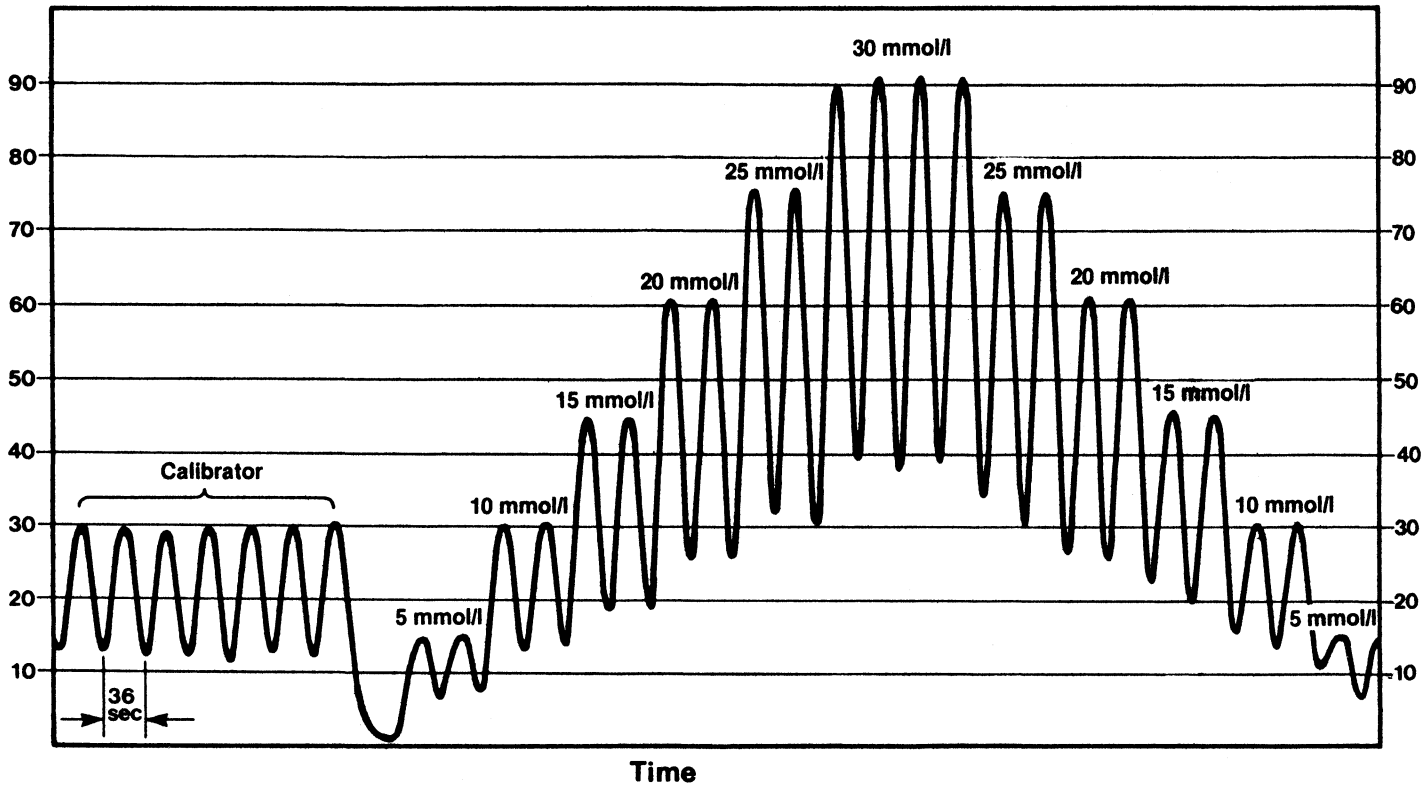

*Figure 4. Recorder chart's print-out for linearity of the calibration function. The linearity is as demonstrated on the figure with a production rate of 100 determinations/h and a sample-to-wash ratio of 2 : 1. Age of enzyme coil: 14 days. Calibrator: 10 mmol/l = 30 recorder units. Calibrator: 30 mmol/l = 90 recorder units. Dilution of 30 mmol/l calibrator with 0·154 mol/l NaCl. 10 recorder units are equal to an absorbance of 0·02.*

20 mmol/l is an adequate linearity limit for samples in clinical chemistry. With a daily sample load of 80, it was found that two samples had to be reanalysed after dilution. This is acceptable, especially because the number of enzyme coils used per year can then be reduced from 12 to four: this will save about $1500/year.

### *Precision*

The precision (within series) was calculated as a coefficient of variation by means of duplicate determinations. The figure also includes the imprecision of sample preparation: C.V. = 2·0% (the mean, $\bar{x}$ = 4·63 mmol/l, numbers of pairs = 48).

### *Drift*

The drift when analysing patient samples is less than 0·20 mmol/l during 40 determinations and less than 0·22 mmol/l for a serum control sample during 40 determinations.

### *Sensitivity*

The sensitivity (i.e. the increase in absorbance due to a glucose concentration of 1 mmol/l) is $0{\cdot}015\ (^{mmol}/l)^{-1}$.

### *Normal range*

The normal range was determined with 48 healthy, fasting people (children and adults). Distribution of the results was found to be Gaussian with a mean ($\bar{x}$) of 4·63 mmol/l, and a standard deviation (SD) of 0·35 mmol/l. The normal range was calculated as $\bar{x} \pm 2$ SD: 3·9 mmol/l $\leqslant x \leqslant$ 5·3 mmol/l ($p = 0{\cdot}95$). This is in accordance with the normal range of the glucose dehydrogenase (DH) method: 3·3 mmol/l $\leqslant x \leqslant$ 5·5 mmol/l ($p = 0{\cdot}95$).

### *Economy*

See figure 5.

*Figure 5. Expected economy.*

1. 300 determinations performed each day (in series of 10–20 determinations).
2. Tubes and membranes changed weekly.

| | |
|---|---|
| Working reagent<br>Saline and water with Brij-35 | $/year<br>2000 |
| Enzyme coils | 800 |
| Disposables | 750 |
| Annual cost | $3550/year |

### Comparison with the glucose DH method

Figure 6 shows the results on 106 samples of whole blood (duplicates), determined with the present method and with the glucose DH method—this is a method known to give accurate results [1 and 2]. There is an average deviation between the two methods of 5%, calculated by linear regression. The glucose DH method gives the highest values, perhaps because the proteins and erythrocytes in the sample are precipitated, resulting in a minor volume in which the amount of glucose is dissolved, i.e. the concentration will increase. The coefficient of correlation is r = 0·9921.

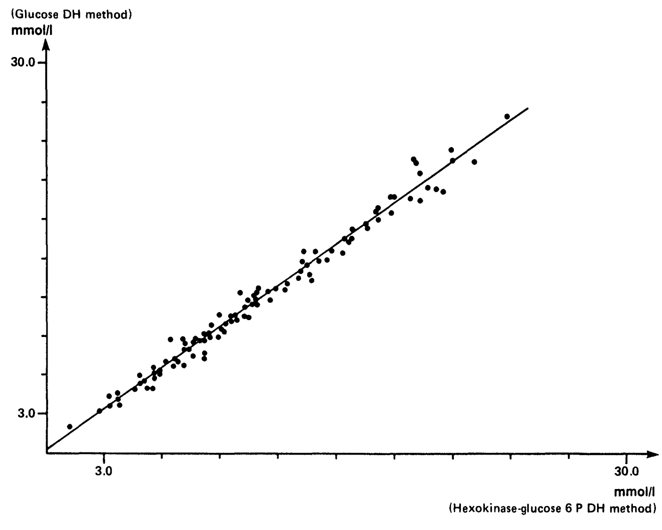

*Figure 6. Comparison of the results on whole blood by the glucose dehydrogenase (DH) method (y) (materials: capillary, heparinized blood, precipitated with perchloric acid) and the immobilized hexokinase-glucose 6 P dehydrogenase (DH) method (x) (materials: capillary, heparinized blood, diluted with heparin, NaF, benzoic acid). Where y (gluc. DH) = 1·05·x + 0·3; $S_{y/x}$ = 0·69 mmol/l; correlation coefficient, $r_s$ = 0·9921; number of determinations, N = 106; $\bar{y}$ = 11·88 mmol/l; and $\bar{x}$ = 11·04 mmol/l.*

Figure 7 shows the results on 26 samples of urine (duplicates) determined with the present method and with the glucose DH method. There is an average deviation between the two methods of 3%, calculated by linear regression. Again the glucose DH method gives the highest values, perhaps due to protein or leucocytes. The coefficient of correlation is r = 0·9971.

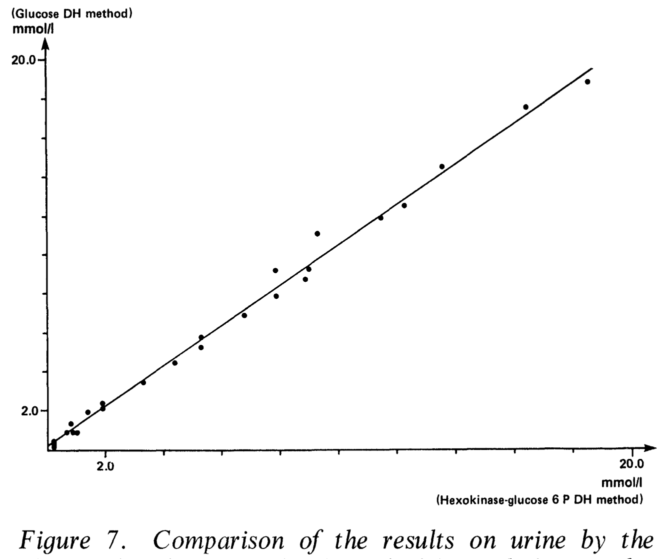

*Figure 7. Comparison of the results on urine by the glucose dehydrogenase (DH) method (y) and the immobilized hexokinase-glucose 6 P dehydrogenase (DH) method (x). Where y (Gluc.DH) = 1·03·x + 0·2; $S_{y/x}$ = 0·43 mmol/l; correlation coefficient, $r_s$ = 0·9971; number of determinations, N = 26; $\bar{y}$ = 6·15 mmol/l and $\bar{x}$ = 5·79 mmol/l.*

### Conclusions

The described modifications have a number of advantages in comparison to the original method: SE 40046FD9 (Technicon). The use of immobilized enzymes is an attractive feature, but makes the analytical system very sensitive to large changes in pH when it is being cleaned. If immobilized enzymes are to be introduced into a multichannel analytical system, which is used day and night by different technicians, it is necessary to protect the enzyme coil with solenoid valves connected to a controller. The controller is connected to the device so that all handling is automatic. The settings of the solenoid valves are changed by the controller when the last sample has left the system so that the buffered coenzyme reagent can be recirculated, resulting in a reduction in reagent costs. If the analytical system is of the single-channel type the three reagent lines are equipped with one-way valves so that the flow can be immediately stopped by unlatching the cover plate and platen of the proportioning pump without back-flush of reagents. In this way the bubble pattern is undisturbed making it possible to stop and start analysing without breaking the pattern. This also leads to a reduction in reagent costs.

The linearity of the calibration function is better than, or equal to, 20 mmol/l over three months. The stability of the buffered coenzyme reagent is one month at room temperature, in comparison with Technicon's week. By using 300 $\mu$l heparin, NaF, benzoic acid as diluent (in a 400 $\mu$l polyethylene tube) when preparing the whole blood/urine from sample (in a 20 $\mu$l heparinized capillary), precipitation of proteins and erythrocytes are avoided as well as centrifugation. All samples can then be put directly into the sampler and analysed, giving a response time of less than 4 min.

The results on whole blood/urine deviate by ÷5%/÷3% when compared with the glucose DH method.

According to Leon *et al.* [1] the accuracy of the present method is excellent. With a load of 300 determinations/24 h in series of 10–20, and changing of tubes and membranes weekly the annual costs are estimated to be $3550 a year.

### Acknowledgements

Anders and Else Sørensen of the Insulinlaboratoriet, Novo Industries, Copenhagen are thanked for advice about the evaluation. The authors are also grateful to Nina Friis and Alice Andersen for skilful technical work.

### References

1. LEON, L. P., CHU, K. D., SNYDER, L. R., and HORVATH, D., *Clinical Chemistry*, **26** (1980), 123.

2. BANAUCH, D., BRÜMMER, W., EBELING, W., METZ, H., RINDFREY, H., LANG, H., LEYBOLD, K., and RICK, W. Z., *Klin. Chem. u. Klin. Biochem.*, **13** (1975), 101.

### ROYAL SOCIETY OF CHEMISTRY, ANALYTICAL DIVISION

*Programme of Meetings for the Session 1983–84*

A booklet listing the conferences and meetings of the various regions and groups is available from the Society (Burlington House, London W1V 0BN, tel.: 01 734 9971). Current secretaries of the Division's groups are:

*Microchemical Methods Group*—P. R. W. Baker, Department of Physical Chemistry, Wellcome Research Laboratories, Langley Court, Beckenham, Kent BR3 3BS, UK.

*Special Techniques Group*—J. Huddlestone, AERE Building 10.2, Instrumentation and Applied Physics Division, Harwell, Oxfordshire OX11 0RA, UK.

*Biological Methods Group*—A. H. Thomas, National Institute for Biological Standards and Control, Holly Hill, Hampstead, London NW3 6RB.

*Atomic Spectroscopy Group*—D. J. Willis, Hilger Analytical, Westwood, Margate, Kent CT9 4JL, UK.

*Chromatography and Electrophoresis Group*—Diana Simpson, Analysis for Industry, Factories 2/3, Bosworth House, High Street, Thorpe-le-Soken, Essex CO16 0EA, UK.

*Thermal Methods Group*—C. J. Keattch, Industrial and Laboratory Services, PO Box 9, Lyme Regis, Dorset, UK.

*Automatic Methods Group*—C. J. Jackson, Occupational Hygiene Laboratory, 403 Edgware Road, London NW2 6LN.

*Particle Size Analysis Group*—J. E. C. Harris, Materials Quality Assurance Directorate, Ministry of Defence, Puriton, Bridgwater, Somerset TA7 8AD, UK.

*Radiochemical Methods Group*—M. A. Crook, London School of Polymer Technology, Polytechnic of North London, Holloway Road, London N7 8DB.

*Electroanalytical Group*—A. E. Bottom, Kent Industrial Measurements Ltd, EIL Analytical Instruments, Hanworth Lane, Chertsey, Surrey KL16 9LF, UK.

*Education and Training Group*—L. A. Gifford, Department of Pharmacy, University of Manchester, Oxford Road, Manchester M13 9PL, UK.

*Joint Pharmaceutical Analysis Group*—B. V. Fisher, The Wellcome Foundation Ltd, Central Analytical Laboratories, Temple Hill, Dartford, Kent DA1 5AH, UK.

The Automatic Methods Group's forthcoming activities are listed in this quarter's Calendar.

